# Cost benefits of cardiovascular magnetic resonance imaging and angiography performed earlier in the diagnostic assessment of neonatal complex congenital heart disease

**DOI:** 10.1186/1532-429X-16-S1-O97

**Published:** 2014-01-16

**Authors:** Pierangelo Renella, Derek Phan, Sarah N Khan, Brian Reemtsen, J Paul Finn, Gary M Satou

**Affiliations:** 1 Radiology, David Geffen School of Medicine at UCLA, Los Angeles, California, USA; 2 Pediatrics, David Geffen School of Medicine at UCLA, Los Angeles, California, USA; 3 David Geffen School of Medicine at UCLA, Los Angeles, California, USA; 4 Pediatric Cardiothoracic Surgery, David Geffen School of Medicine at UCLA, Los Angeles, California, USA

## Background

Neonates with complex congenital heart disease (CCHD) often require transcatheter or surgical intervention in the first month of life. Transthoracic 2D/Doppler echocardiography (2DE) is the diagnostic modality of choice, but occasionally may be inadequate for surgical planning. Unresolved pre-operative questions may require complete delineation of extracardiac vascular anatomy and/or ventricular volumes. In these cases, cardiac magnetic resonance imaging and angiography (CMR/MRA) may be delayed in favor of repeat 2DE studies, which often remain inconclusive. This approach may lead to unnecessary costs and has yet to be formally evaluated.

## Methods

A retrospective review of neonates with CCHD who underwent CMR/MRA (2007-12) was performed to compare the costs of imaging between two groups of patients: one undergoing CMR/MRA after the initial 2DE but prior to any additional 2DE studies (Group A), and those with multiple 2DE studies prior to CMR/MRA (Group B). Thirty-one patients were identified (median age at CMR/MRA was 4 days; range 2-25 days). Five patients were excluded (three without CHD; two without 2DE prior to CMR/MRA). The number of additional 2DE studies (after the initial 2DE) performed prior to CMR/MRA in Group B as compared to Group A ranged from one to three. A single CMR/MRA study prior to surgical intervention was obtained in all patients. All CMR/MRA studies were performed under general anesthesia (provided by a neonatologist) without adverse events.

## Results

The total cost of cardiac imaging (2DE and CMR/MRA) in Group A (n = 12) was $26,896 (2DE = $14,603; CMR/MRA = $12,293). The total cost in Group B (n = 14) was $50,495 (2DE = $36,153; CMR/MRA = $14,342). In total, $23,599 less was spent in Group A as compared to Group B, with a mean per patient cost savings of $1,366 between the two imaging strategies.

## Conclusions

CMR/MRA has been shown to be of incremental benefit in the preoperative assessment of CCHD. However, depending on practice patterns and/or local availability of CMR, a diagnostic approach using multiple/serial 2DE studies is often utilized. This study is the first to suggest that a strategy involving the earlier use of CMR/MRA in the diagnostic algorithm of CCHD patients may improve cost efficiency and cost savings. Further investigation that includes quantification of the diagnostic power of both 2DE and CMR/MRA across several congenital heart disease states may better define whether earlier and more routine use of CMR/MRA is cost effective.

## Funding

None.

**Table 1:** Patient Diagnostic Categories

	Number of Patients (n = 26)
Anomalous Pulmonary Venous Connection	1
Coarctation of the Aorta	4
Conotruncal Defects	10
Double Inlet Left Ventricle	1
Heterotaxy syndrome	2
Tricuspid Atresia	2
Multiple Left-Sided Obstructive Lesions	4
Vascular Ring	2

**Figure 1:**
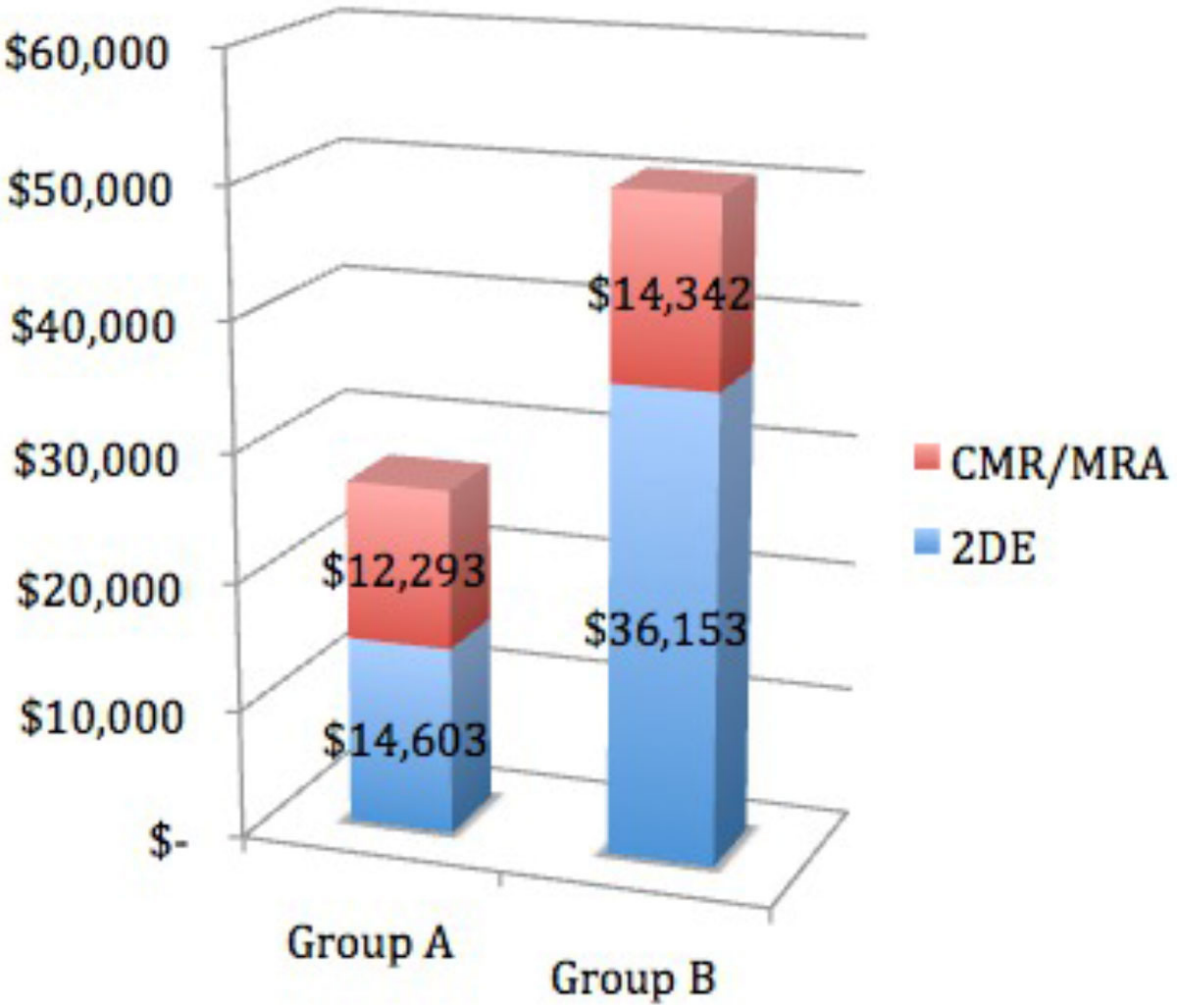
**Cardiac Imaging Cost Comparison Between Groups A and B**.

